# A multicentred two-arm parallel single-blind superiority randomised controlled trial comparing psychological and emotional stabilisation with eye movement desensitisation and reprocessing and treatment-as-usual to treatment-as-usual with adults with intellectual disabilities who have post-traumatic stress disorder (the Trauma-AID trial): protocol

**DOI:** 10.1136/bmjopen-2025-108818

**Published:** 2025-12-17

**Authors:** Paul Willner, Pippa Anderson, Jonathan I Bisson, Clair Clifford, Vivien Cooper, Derek Farrell, Shaun Harris, Stephen Hiles, Gail Holland, Hayley Anne Hutchings, Glynis H Murphy, John Rose, Biza Stenfert-Kroese, Gemma L Unwin, Alan Watkins, Sara Willott, Peter E Langdon

**Affiliations:** 1 School of Psychology, Swansea University, Swansea, UK; 2 Swansea Trials Unit, Swansea University, Swansea, UK; 3 DIvision of Psychological Medicine and Clinical Neurosciences, Cardiff University, Cardiff, UK; 4 Clifford Psychological Services, Solihull, UK; 5 Challenging Behaviour Foundation, Chatham, UK; 6 Northern Hub for Veteran and Military Families' Research, Northumbria University, Newcastle upon Tyne, UK; 7 Tizard Centre, University of Kent, Canterbury, UK; 8 School of Psychology, University of Birmingham, Birmingham, UK; 9 Birmingham Community Healthcare NHS Foundation Trust, Birmingham, UK; 10 Intellectual Disabilities Research Institute (IDRIS), University of Birmingham, Birmingham, UK; 11 Herefordshire and Worcestershire Health and Care NHS Trust, Worcester, UK

**Keywords:** MENTAL HEALTH, Clinical Trial, Adult psychiatry

## Abstract

**Introduction:**

The primary objective of this clinical trial is to determine the clinical and cost-effectiveness of psychoeducation and emotional stabilisation (PES), together with eye movement desensitisation and reprocessing (EMDR) plus treatment-as-usual (TAU) in reducing symptoms of post-traumatic stress disorder (PTSD) among adults with intellectual disabilities compared with TAU. Secondary objectives include: (1) determining whether PES/EMDR plus TAU is superior to TAU in improving mental health problems and quality of life (QoL) among adults with intellectual disabilities who had a diagnosis of PTSD and (2) completing a process evaluation to examine intervention implementation and acceptability.

**Methods:**

This is a two-arm parallel single-blind randomised controlled trial comparing PES-EMDR+TAU to TAU including an internal pilot phase. Outcome data will be captured prior to randomisation, and at 4 (after PES), 8 (after EMDR) and 14 months postrandomisation by masked assessors. 144 adults with intellectual disabilities with a diagnosis of PTSD will be allocated (1:1) randomly using minimisation from National Health Service (NHS) community and inpatients services for adults with intellectual disabilities in England. Participants are eligible to take part in this trial if: (1) they are aged 18 or older, but younger than 66, (2) have a Full Scale IQ<75, (3) meet diagnostic criteria for PTSD and (4) have suffered a major identified trauma at least a year earlier and (5) are able to communicate using English and have capacity to consent to take part in this clinical trial. Participants allocated to the active intervention will receive 10 sessions of PES, followed by up to 15 sessions of EMDR alongside TAU. The active intervention is being delivered by psychologists experienced in working with adults with intellectual disabilities who have received additional intervention training. TAU is likely to include medication, behaviour support plans designed to target challenging behaviour, or non-trauma-focused psychological interventions. The primary outcome is a measure of PTSD symptoms. Secondary outcomes are other mental health problems, including anxiety and depression, challenging behaviour, participant and carer QoL, and carer burden. We are also capturing cost data to allow for a cost–utility analysis. A process evaluation will be completed using data generated from semistructured interviews with a sample of participants, therapists and carers alongside the capture of fidelity and adherence data.

**Analysis:**

The primary outcome will be assessed using an intention-to-treat analysis. Baseline characteristics will be compared between arms to determine whether any potentially influential imbalance occurred. The primary outcome will be analysed by analysis of covariance, adjusting for baseline values of the outcome and any variables used in the randomisation process. Secondary outcomes will be analysed using linear or logistic regression models as appropriate reflecting the distribution of the outcome variable. The treatment effect will be estimated as an adjusted difference between sample means, presented with 95% CIs and p values. A complier average causal effect analysis will be considered should the data availability be sufficient to estimate the impact of non-compliance. A series of subgroup analyses on the primary outcomes will be considered considering differences in the Impact of Event Scale–Intellectual Disabilities scores at 14 months for (1) differing levels of general intellectual functioning and (2) PTSD versus complex PTSD.

**Ethics and dissemination:**

This clinical trial was designed to allow for conclusions about whether PES/EMDR+TAU is efficacious in reducing symptoms of PTSD, relative to TAU, for adults with intellectual disabilities. A favourable ethical opinion has been received from an NHS ethics committee in the UK. The findings from this trial will be published within peer-reviewed journals and shared at national and international conferences. We will also aim to record and distribute podcasts detailing our findings together with our partners.

**Trial registration number:**

ISRCTN35167485.

**STRENGTHS AND LIMITATIONS OF THIS STUDY**

- The evidence to support the use of psychological therapies with people with intellectual disabilities is limited; this clinical trial will help address this problem by generating evidence about the effectiveness PES/EMDR plus TAU relative to TAU in reducing symptoms of PTSD.
- People with intellectual disabilities face discrimination and stigma which perpetuate their social exclusion including their exclusion from clinical trials. Our clinical trial will also help address this problem.
- Initially, the trial was severely impacted by the COVID-19 pandemic but recovered. Participants are currently in follow-up, and we are on track to report in accordance with our revised timeline.
- We are unable to provide treatment via an interpreter for those who do not speak English.

## Post-traumatic stress disorder in people with intellectual disabilities

 Post-traumatic stress disorder (PTSD) is a common mental disorder that may develop following exposure to traumatic events. About 3% of the adult population in England suffers from current PTSD^1^ and a lower IQ is associated with increased rates of PTSD.^2^ There is extensive evidence that people with intellectual disabilities are 4–6 times more likely than the general population to suffer severe and prolonged bullying and/or sexual and other types of abuse,^3^,^5^ and adverse life events are traumatising in this population.^6^,^8^ Exposure to trauma is known to impair executive functioning^9^ and the impact of this loss of cognitive resources may be exacerbated, and risk heightened, for those with a developmental disability who may find coping difficult. It is no surprise that rates of PTSD are higher in people with intellectual disabilities than in the general population.^2^

PTSD has simple and complex presentations. Simple PTSD typically follows a single traumatising event such as a road traffic accident, while complex PTSD typically follows a history of chronic traumatisation such as prolonged abuse.^5 10^ In addition to the characteristic symptoms of PTSD (re-experiencing, avoidance and hyperarousal), complex presentations of PTSD include further symptoms arising from a disturbance of self-regulatory capacities resembling aspects of borderline personality disorder (difficulty in regulating emotions; feelings of shame, guilt and worthlessness; difficulties in sustaining relationships and feeling close to others).^10^ Recently, the International Classification of Disease (ICD) diagnostic system recognised complex PTSD as a separate diagnosis.^5^

A relatively recent study of people with intellectual disabilities about treatment of PTSD reported that almost all had experienced multiple traumatic events in adulthood and around half (probably an underestimate) reported that they had also experienced traumatic events in childhood.^11^ People with intellectual disabilities who have been traumatised typically show complex presentations of PTSD and display physical and psychiatric comorbidity, as well as self-harm or other challenging behaviours,^4 12^ particularly those with autism.^13^ Frequently, PTSD is not diagnosed among this group, and treatment focuses on the management of challenging behaviour. These patients are extremely complex and display behaviours that carers and services experience as challenging. They require highly specialist National Health Service (NHS) intellectual disability services, considerable community support and are at risk of admission to hospital. Their symptoms cause them and those around them significant distress.

## Treatment of PTSD

On the basis of evidence from systematic reviews and meta-analyses, the UK National Institute for Health and Care Excellence (NICE) and other clinical guidelines recommend trauma-focused psychological therapies for PTSD,^14 15^ since therapies that do not require the patient to focus on traumatic memories are less effective.^16^ The best-supported trauma-focused interventions are trauma-focused cognitive behavioural therapy (TF-CBT) and eye movement desensitisation and reprocessing (EMDR).^16^ EMDR can include an initial phase of PES, before the main treatment phase in which the patient focuses on memories of past traumatic events while making controlled eye movements (or an alternative form of bilateral stimulation) that engage attention and reduce the vividness and emotionality of recalled memories.^17^,^19^

While there is little to choose between TF-CBT and EMDR in terms of their effectiveness for reducing PTSD symptoms, they differ in the experience offered to the patient. TF-CBT is a talking psychological therapy that aims to identify and modify overinterpretations of the actual level of threat, and to modify beliefs and interpretations regarding the traumatic event; verbal communication is critical to change. By contrast, EMDR is less reliant on talking during therapy as the patient attends to emotionally disturbing material, in brief episodes, while simultaneously focusing on an external stimulus, typically, therapist-directed bilateral eye movements^20^; bilateral stimulation is considered critical to change. EMDR is typically described as involving eight phases: (1) history taking and treatment planning; (2) preparation; (3) assessment; (4) desensitisation; (5) reprocessing, which involves installing positive cognitions; (6) body scan; (7) closure and (8) reassessment. The trauma confrontation work begins in phase 4.

There has been relatively little research on interventions for more complex presentations of PTSD, but what evidence there is suggests that phased approaches may be beneficial, in which the patient first undergoes PES before undertaking any trauma-focused intervention.^10 21^ The PES phase targets problems such as affect dysregulation, interpersonal relationships, dissociation and somatic symptoms, so as to promote adaptive coping, a sense of safety and emotional stabilisation. PES includes the first phase of the EMDR protocol (history taking and treatment planning) and aspects of phases 2 and 3 (preparation and assessment) and could lead on either to TF-CBT or to the later phases of EMDR. However, there is also some evidence that a stabilisation phase prior to EMDR does not improve treatment outcomes.^22^

## EMDR for people with intellectual disabilities

It was thought for many years that people with intellectual disabilities could not benefit from psychotherapy, leading to decades of neglect described as ‘the unoffered chair’.^23^ Over the past 30 years,^24^ this assumption has been increasingly challenged.^25^,^30^ However, the evidence that exists is biased due to a preponderance of small and poorly designed studies with very few randomised controlled trials making conclusions about effectiveness fraught.^25^ Large, well-designed clinical trials are needed to ensure that psychological therapies for mental health disorders are effective for people with intellectual disabilities, as this group has a high level of need but is all too often excluded from clinical research.

EMDR is recommended by NICE, and internationally, as a first-line treatment of choice for PTSD in the general population.^15 31^ EMDR is a relatively simple procedure that is ostensibly less reliant than CBT on verbal communication which may be more suited to people with intellectual disabilities. There are a number of case study reports^32^,^34^ and one small controlled trial^35^ providing weak evidence that adapted EMDR protocols can be used to treat PTSD in people with intellectual disabilities.

## Complex PTSD

The high prevalence of complex presentations of PTSD among people with intellectual disabilities has meant that this issue needed careful consideration in relation to diagnosis, assessment and the design of the intervention for people with intellectual disabilities. These considerations have influenced three aspects of this trial:

1. Clinical trials of treatments for PTSD have typically used Diagnostic and Statistical Manual of Mental Disorders (DSM) criteria or ICD criteria which is used in the NHS and the UK. The current version, ICD-11, is advantageous because PTSD and complex PTSD are separate disorders, with additional criteria for complex PTSD that can be used to estimate the degree of complexity.^36 37^ By contrast, DSM-5 does not distinguish PTSD and complex PTSD, creating uncertainty over the extent to which treatments are effective for either presentation. In DSM-5, a complex presentation is loosely indicated by the qualifier ‘with dissociative features’, but it is difficult, often impossible, to assess whether dissociative features are present in people with intellectual disabilities. ICD-11 focuses on disturbances in self-organisation, including underlying emotional lability and distress, which can be readily assessed. There is also evidence that a PTSD diagnosis using ICD-11 criteria has greater validity than DSM-5.^38^ For all these reasons, we are using ICD-11 criteria to diagnose PTSD, rather than DSM-5. The ICD-11 beta draft has been available online since 2015: the version for implementation was published in 2018 and implemented in 2022 5. The International Trauma Questionnaire (ITQ) was designed to provide independent assessment of ICD-11 PTSD and complex PTSD.^36 37^ In a preliminary study, we validated a version of the ITQ adapted for people with intellectual disabilities.^39^
2. EMDR protocols for PTSD and Complex PTSD start with a preparatory PES phase before commencing desensitisation and reprocessing procedures.^20^ For complex PTSD, both NICE and expert consensus guidelines recommend that the two phases of treatment are considered separately, with a common preparatory PES phase, followed by a choice of approaches (EMDR or TF-CBT) thereafter.^21 40^ Because we anticipate that a high proportion of patients will display complex presentations of PTSD, and because, in our experience, people with intellectual disabilities require more extensive preparatory work, our treatment package begins with a free-standing PES module.
3. A secondary aim of the trial is to achieve a clearer understanding of whether the clinical efficacy of EMDR is related to the degree of complexity in PTSD presentation. A picture is emerging from the general literature that EMDR may be less effective for chronic and complex presentations of PTSD.^41^,^43^ The sparsity, methodological diversity and generally poor quality of the literature preclude any conclusion about whether this would apply to people with intellectual disabilities with PTSD. Use of the ICD-11 diagnostic criteria, rather than DSM-5, enables measurement of the complexity of PTSD presentation,^36 37^ which therefore can be included as a factor in the analyses, alongside demographic variables such as age, gender and Full-Scale IQ. These analyses will also reveal whether the Full-Scale IQ is related to the efficacy of EMDR.

## Risks and benefits

We have judged that there are no significant risks to participants or society. There is a hypothetical risk that a patient could temporarily worsen during therapy, which is not uncommon and may precede clinical improvement. However, this has not been identified as a significant issue in the relevant literature.

A potential benefit to participants is that they may learn to cope better with their traumatic memories, with a concomitant decrease in symptoms of PTSD including challenging behaviour, so increasing their opportunities for social inclusion and decreasing the risk of placement breakdown, reliance on potentially dangerous medications, exclusion from services and involvement with the criminal justice system. A potential benefit to society is the avoidance of these outcomes, which are costly to services and impinge on other service users and members of the public. There are also potential benefits to carers and families, in relation to decreased occupational/family stress and improved social relationships.

## Patient and public involvement

The trial is supported by three patient and public involvement (PPI) panels: a group of people with intellectual disabilities who have experience of supporting research; a group of male service users with intellectual disabilities, the majority of whom have experienced, and been treated for, trauma; and a group of carers of adults with intellectual disabilities and PTSD. Additionally, two members of the Trial Steering Committee are senior officers within third-sector organisations supporting people with intellectual disabilities. They will be involved in reviewing the analysis of data and dissemination of results through their third-sector organisations and they will be acknowledged for their contributions in trial outputs.

## COVID-19

The COVID-19 pandemic caused severe disruption to the delivery of healthcare globally. Planned recruitment to this trial was temporarily halted during the pandemic and then started in 2022. In order to provide a safe and robust framework to allow the trial to operate under what were historical COVID-19 restrictions, we explored the feasibility of remote working and a hybrid model in which therapy would usually commence face-to-face, but therapists would be able to switch to a remote platform if it became necessary to discontinue face-to-face contact (Unwin *et al*).^44^

### Objectives

The primary objective of the trial is to determine the clinical and cost effectiveness of PES/EMDR+TAU relative to TAU in adults with intellectual disabilities.

Secondary objectives are:

- To determine whether PES/EMDR leads to improvements in other mental health problems and QoL.
- To conduct an economic evaluation of PES/EMDR, relative to TAU, with people with intellectual disabilities.
- To evaluate patient and carer satisfaction with PES/EMDR.
- To determine whether outcome following treatment with PES/EMDR is related to the complexity of PTSD.

### Trial design

This is a two-armed parallel single-blind multicentre randomised controlled clinical trial comparing EMDR/PES+TAU to TAU including an internal pilot. The participant allocation ratio is 1:1.

The internal pilot phase is 18 months and has the following objectives: (a) recruit 36 participants by 14 months and (b) complete the intervention with at least 6 participants by 18 months to test adherence and retention through the two phases of the intervention. Progress during the internal pilot phase will be monitored by the Trial Steering Committee (TSC) and the Data Monitoring and Ethics Committee (DMEC) who will report directly to the funder. Any difficulties in meeting these objectives will be reviewed, and if possible, an appropriate mitigation strategy will be developed and implemented.

### Methods: participants, interventions and outcomes

#### Trial setting

The trial will be located in NHS hospital and community services for people with intellectual disabilities. They are listed in online supplemental material: appendix 1.

### Eligibility criteria

#### Inclusion criteria

Adults with intellectual disabilities

- Aged ≥18 to ≤ 65.
- Meeting criteria for a diagnosis of intellectual disability confirmed by: (a) existing diagnosis of intellectual disability and in receipt of specialist NHS services for adults with intellectual disabilities, confirmed at screening as having a Full Scale IQ≤75 or (b) completion of an assessment of both level of general intellectual functioning and adaptive behaviour confirming diagnostic criteria for intellectual disability are met with both IQ/composite scores ≤75 .
- Meeting ICD-11 diagnostic criteria for PTSD, as assessed by the International Trauma Questionnaire–Intellectual Disability (ITQ-ID).^39^ A diagnosis of PTSD requires the presence of symptoms from each of the three PTSD symptom clusters and evidence of functional impairment. For the current trial, a broader definition is used, comprising either the presence of symptoms from two PTSD symptom clusters plus functional impairment, or the presence of symptoms from all three clusters with no declared functional impairment.
- Major identified trauma at least a year earlier.
- Able to communicate in English and has the capacity to decide whether they wish to take part in the trial.

##### Carers

Patients should ideally have a carer who can participate, but those who do not will be included. Carers will be invited to take part if they fulfil these inclusion criteria:

- Aged 18 and over.
- A family member or carer of a person with intellectual disabilities who has consented to participate in the trial.
- Able to communicate in English and has the capacity to decide whether they wish to take part in this trial.
- Able to attend clinic visits (or remote sessions if offered) or be present when a researcher performs the assessment visit.

Carers are defined as ‘the primary person who feels responsible for and provides support to the person with intellectual disability on a regular basis as judged by a clinician’. In the situation where the carer attending subsequent assessment visits is different from previous ones, they will be asked to consent, to minimise missing data.

### Exclusion criteria

- Assessed by the clinical team as at high risk and/or requiring urgent treatment.
- Currently in therapy and unwilling to intermit.
- Previously completed a course of EMDR.
- Psychosis not well controlled by medication.
- Change of psychotropic medication or dosage within the last month.
- Unable to complete the assessments.
- Any medical condition or treatment which, in the opinion of investigators, could affect the safety of the patient or outcomes of the study.

### Consent procedures

Potential participants will be identified by the clinical team to which they have been referred on the basis of an assessment of their trauma history. Where there is evidence to suggest that eligibility criteria are met, information about the trial will be shared with a potential participant. Consent will be taken by a member of the research team.

A standard research consent procedure will be used with patients, in which:

- The trial is explained verbally in simple terms to the service user using an Information Script, checking frequently for understanding.
- In addition to the full participant information sheet (PIS), service users are also given a simplified information sheet to take home and read in their own time and at their own speed.
- At least 3 days are given to allow potential participants and/or carers to be asked questions and consider taking part.
- The explanation is repeated in a second meeting.
- Consent is recorded by the researcher reading each paragraph of the consent form and the patient checking and initialling a set of tick boxes and signing the consent form.
- In order to assure that the patient has been properly informed, the whole process may be witnessed by a third party (eg, a carer) who is independent of the research team.
- A narrative account of the process, along with relevant documents and files, is added to the local clinical note system.

A similar procedure is followed if remote consent is needed; it can be evidenced by means of a video or screenshot. The full remote consent procedure is listed as online supplemental material: appendix 2.

Similar consent procedures will be used with carers. Copies of our participant and carer trial information sheets and consent forms are found within online supplemental material: appendix 3.

### Interventions

#### Explanation for the choice of comparators

The comparator for the trial is TAU which will be as defined by the therapist and could include any non-trauma-focused intervention. When the trial was designed, trauma-focused interventions were rarely used in the UK with people with intellectual disabilities. They have since become more common, but the restriction was maintained in order to avoid introducing into the trial an element of comparison between two active trauma-focused treatments.

#### Intervention description

The standard EMDR protocol is difficult to use with people with intellectual disabilities because the eye movement exercises (or alternative bilateral stimulation procedures) are unfamiliar, and their purpose is difficult to explain. However, the procedure can be made more accessible for people with intellectual disabilities and acceptable to therapists by expanding the introductory PES phase and using some of the techniques developed for use with traumatised children (but adapted so as to be appropriate for adults).^45^ Some case study reports suggest that adapted EMDR protocols can be used to treat PTSD in people with intellectual disabilities.^32 33^ However, in our experience, therapists do not feel comfortable using the standard EMDR protocol with people with intellectual disabilities, as clients find it difficult to understand the rationale and the terminology and to manage the desensitisation and reprocessing stages.^46^ We also find that people with intellectual disabilities need extensive preparation before commencing EMDR in order to increase engagement (distrust of services and not being listened to being common experiences) to ensure that they have sufficient understanding of what they need to do, and why, and to militate against dropout from the EMDR phase.

This trial will therefore use a bespoke EMDR protocol that includes, as phase 1, a PES module that aims to instal strengths and resources, stabilise emotional regulation, and build alliance and trust,^40^ and in phase 2 incorporates elements of the EMDR protocol as adapted for children,^47^ with some changes to make it age appropriate for people with intellectual disabilities.

Phase 1 is a 10-session PES protocol that has been previously adapted for people with intellectual disabilities from a PES protocol used routinely with patients with PTSD in some adult mental health services.^48^ Both therapists and people with intellectual disabilities in treatment find PES acceptable. Participants have previously provided positive feedback about their experiences of taking part in our PES protocol.^48^ Although piloted in a group format, the PES module can readily be delivered on an individual basis.^48^ For the purposes of this trial, the PES module was further adapted by including an introduction to bilateral stimulation.

We have also previously piloted a modified phase 2 EMDR protocol.^46^ The major adaptations^45^ are (1) making the stages, language and outcomes more accessible; (2) not preferring side-to-side finger movements over other forms of bilateral stimulation such as tapping; (3) encouraging creative use of expression (such as techniques from art and narrative therapy/storytelling eg,^47^ and (4) involvement of carers where appropriate to support the patient within and/or between therapy sessions.

The intervention is fully manualised. The PES phase comprises 10 weekly sessions which can be extended if required, while the EMDR phase also comprises 10 sessions but can be extended to 14 sessions if required.

Within each trial site, therapists have been trained to deliver PES/EMDR. Training comprised the standard accredited training (previously known as levels 1 and 2 and currently known either as parts 1–3 or parts 1–4). It followed the standard training curriculum split over two blocks to include a further day on our adapted protocol and a final training day following 4–6 months of supervised practice. It is equivalent to standard EMDR accredited training. In light of experience in the early stages of the trial, and in line with the national EMDR training curriculum, therapists are encouraged to undergo 1 month of supervised practice. For a minority of therapists who had previously been trained to deliver EMDR, only the training in our adapted protocol was delivered. All therapists also undertook training on remote delivery of EMDR, comprising a 1-day workshop and access to remotEMDR software (https://www.remotemdr.com/). Additional refresher training was available for sites that experienced significant delays in starting due to COVID-19. The therapists subsequently have access to supervision sessions via MS Teams in small groups, with additional phone supervision available as needed.

#### Criteria for discontinuing or modifying allocated interventions

A participant or their carer may terminate their participation in the trial at any time without giving a reason and with no personal disadvantage. Participants allocated to PES/EMDR may wish to terminate treatment. If this occurs, participants will be invited to remain in the trial and provide outcome data. Trial participants may also be withdrawn by the study team due to serious adverse events (eg, hospitalisation, serious illness, death). The sponsor has the right to terminate this trial at any time. In terminating the trial, the sponsor and the chief investigator will ensure that adequate consideration is given to the protection of trial participants. If the trial is suspended or terminated for safety reasons, the sponsor will promptly inform the chief investigator. The sponsor will also promptly inform the relevant regulatory authorities of the suspension/termination and of the reasons for this action, including the Trial Management Committee and Data Monitoring and Ethics Committee.

#### Fidelity and adherence

Fidelity of treatment delivery will be monitored from encrypted audio recordings of PES and EMDR sessions. For each therapist, one session from each phase will be recorded—with participant consent. Assessment of the PES session will be made using the Trauma-AID PES Intervention Checklist. Some generic items in the checklist were adapted from the Manualised Group Intervention Check;,^49^ a 30-item monitoring instrument for group CBT adapted for people with ID. Additional items were incorporated to reflect manual-specific activities and processes in the PES stage to produce an 18-item checklist that addresses engagement skills, accessibility of presentation, understanding, session content, establishing internal safety/emotional stabilisation and developing adaptive coping/preparation for reprocessing. Assessment of the EMDR session will be made using the Fidelity Checklist for Trauma-AID EMDR Sessions, a 21-item checklist incorporating items adapted from the EMDR Fidelity Rating Scale Version 2^50^ as well as manual-specific items. The checklist covers treatment planning and assessment; preparation; calm place exercise, skills and resources; assessment (ahead of trauma confrontation); desensitisation; installation and closure; and future templates. Each recording will be rated by a senior member of the research team, who is familiar with the manualised intervention, using the appropriate checklist. To examine inter-rater reliability, a second rater, who is an expert in EMDR (CC), will double-rate 15% of the recordings. The sample for dual assessment will be randomly selected.

At the end of each session, therapists complete a Clinical Trials Unit online form to report whether the participant attended the session. The therapist also reports the goals for the session and whether or not they were achieved.

#### Relevant concomitant care permitted or prohibited during the trial

Participants can receive any concomitant care considered suitable by their clinical team other than another trauma-focused psychological therapy.

#### Provisions for post-trial care

As participants are treated by clinical services to which they have been referred, those services will remain responsible for post-trial care. Participants will have access to normal NHS complaints and compensation procedures. No special procedures are used.

### Outcomes

#### Primary outcome

- PTSD symptoms. The self-report revised Impact of Event Scale–Intellectual Disabilities (IES-IDs)^6^ is our primary outcome measure. We use the IES-IDs rather than the Clinician Administered PTSD subscales^51^ because the IES-IDs have been adapted and tested for use with people with intellectual disabilities.^6 39^

#### Secondary outcomes

- PTSD symptoms. The self-report ITQ-ID 39 and the informant-version Lancaster and Northgate Trauma Scale^7 52^.
- Mood. The self-report Glasgow Depression and Anxiety Scales ^53 54^ and Clinical Outcomes in Routine Evaluation–Learning Disability.^55^
- Mental Health. Carer ratings of the participant’s mental health (MPAS-ID)^56^ and challenging behaviour, the Aberrant Behaviour Checklist.^57^
- Health-Related Quality of Life (QoL). This is assessed using the self-reported well-being using the Personal well-being Index–Intellectual Disability (PWI-ID)^58^ and carer reports using the Short Form Health Survey (SF-12).^59^ The SF-12 is a standard, well-validated instrument. The PWI-ID is well-validated in general populations and as well as for people with intellectual disabilities. We have included this measure because we feel it is important to attempt to evaluate QoL as experienced by the participants themselves.
- Carer burden. The Warwick-Edinburgh Mental Well-being Scale (WEMWBS).^60^

All outcome measures are completed at baseline and at 4, 8 and 14 months postrandomisation.

### Participant timeline

Our participant schedule of events and schedule of outcome assessments is found within table 1.

**Table 1:** Schedule of events and outcome assessments

	Study period							
	Referral/clinic prescreening* (≤12 weeks)	Screening (<4 weeks)	Baseline Week 0	Phase 1 (PES/TAU) Weeks 5–16		Phase 2 (EMDR/TAU) Weeks 17–27		Follow-up Week 60
Week	**≤–12**	**–4**	**0**	**5–15**	**16**	**17–27**	**28**	**60**
Participant and carer								
Clinical history	Y							
Patient information	Y							
Participant								
Consent		Y						
Diagnostic interview		Y						
Diagnostic review		Y						
Concomitant medication		Y		Y	Y	Y	Y	Y
Confirm eligibility		Y						
Randomisation			Y					
Treatment (1–10 sessions for phase 1 and phase 2)				Y	Y	Y	Y	
Adverse events				Y	Y	Y	Y	Y
Subjective Units of Distress Scale (SUDS)				Y	Y	Y	Y	Y
IQ (WASI-II)		Y						
PTSD History (TIF)		Y						
PTSD Diagnosis/Complexity (ITQ-ID)		Y		Y	Y	Y	Y	Y
Self-reported questionnaires*†			Y		Y		Y	Y
Carer								
Adaptive Behaviour Assessment System - III (ABAS-III)		Y						
Carer-reported questionnaires‡			Y		Y		Y	Y
Self-reported questionnaires§			Y		Y		Y	Y
Service/Support Costs (CSRI-ID)			Y	Y		Y		Y
Audio recording	With participants' consent, one treatment session from phase 1 and phase 2 will be recorded with scoring using the EMDR Fidelity Rating Scale							
Interview	In line with qualitative recruitment methods, around 10 each of patients, carers and therapists will be interviewed about their experiences following completion of therapy.							

All visits are anticipated to take place in clinic or community settings, although other scenarios are permissible as required

* Completed assessments must be within 12 weeks of screening

† Patient completed self-reported questionnaires consist of IES-ID, GDS, GAS, PWI-ID

‡ Questionnaires completed by the carer regarding the participant: MPAS-ID, LANTS, ABC.

§ Questionnaires completed by the carer about their own well-being: SF-12, WEMWBS

ABC, Aberrant Behavior Checklist; CSRI-ID, Client Service Receipt Inventory– Intellectual Disabilities; EMDR, eye movement desensitisation and reprocessing; GAS, Glasgow Anxiety Scale; GDS, Glasgow Depression Scale; IES-ID, Impact of Event Scale–Intellectual Disabilities; ITQ-ID, International Trauma Questionnaire–Intellectual Disabilities; LANTS, Lancaster and Northgate Trauma Scales; PES, psychoeducation and emotional stabilisation; PTSD, post-traumatic stress disorder; SF-12, Short Form Health Survey; TAU, treatment-as-usual; WEMWBS, Warwick-Edinburgh Mental Well-being Scale.

### Sample size

A total of 144 patients will be recruited to the main trial. We aim to detect a medium-to-large effect size (ES) of 0.65, with two-tailed significance at α=0.05 and power=0.90. This requires N=102 independent outcomes, equivalent to N=108 analysable outcomes from small clusters (average of 4 participants per therapist) with an intracluster correlation of 0.02. Our recruitment target thus pragmatically allows for both 25% loss to follow-up and (hitherto unreported) therapist effects in EMDR for PTSD.

A meta-analysis of studies of EMDR versus TAU in the general adult population reported a mean ES=1.17 with 19% loss to follow-up;^16^ a second meta-analysis, restricted to studies of survivors of childhood abuse, which many of our participants are expected to have experienced, reported a smaller ES=0.76.^61^ The mean across these two studies=0.97. For trials of CBT (the only intervention type for which there exists a corpus of information for people with intellectual disabilities), effect sizes for people with intellectual disabilities are small and biased.^25^ The ES used here in sample size considerations is a conservative two-thirds of the mean figure for the two meta-analyses cited.

### Recruitment

The research team meets regularly with principal investigators (PIs) in each of the participating Trusts, and the PIs ensure that their colleagues within clinical psychology and the wider clinical team are alert to opportunities to put forward potential candidates for screening. The recruitment process is overseen by a field coordinator who has close relationships with all PIs. Patients who are newly referred to the service or those on waiting lists will be screened for eligibility to take part.

#### Assignment of interventions: allocation

Potential participants and carers who fulfil all inclusion and meet no exclusion criteria will be informed of their screening results by local research staff and arrangements will be made for randomisation and treatment visits.

A web-based randomisation and back-up system will be provided by Sealed Envelop (https://sealedenvelope.com) based on a trial minimisation algorithm and randomisation list developed in consultation with a Swansea Trials Unit (STU) statistician. Participants will be assigned 1:1 to either the PES+EMDR group or TAU using IQ, PTSD status and gender as minimisation variables. The randomisation protocol will be implemented by the Trial Manager.

#### Assignment of interventions: blinding

Research assistants will be masked to allocation when completing outcome assessments. Given the nature of the intervention, it would be impossible for the clinical team to be blinded. The following safeguards have been implemented to maintain allocation concealment: (1) all PIs and sites have been directed not to discuss or disclose information about therapy to research assistants, (2) trial participants and their carers have been directed not to discuss or disclose information about therapy to research assistants, (3) research assistants have been located away from clinical teams (eg, within NHS Trust Research and Development offices) and (4) should inadvertent unmasking occur, research assistants will report that this has occurred immediately to the Trial Manager. When unmasking occurs, we will replace an unmasked research assistant with a masked research assistant who will then become responsible for data capture.

### Data collection and management

#### Plans for assessment and collection of outcomes

Clinical and health-economic assessments will be conducted by masked assessors, before randomisation and with follow-up at 4 (post-PES), 8 (post-EMDR) and 14 months. The 4-month and 8-month assessments may be delayed by up to a month (or exceptionally, 2 months for the 8-month assessment if the 4-month assessment was delayed); the 14-month assessment will not be delayed. Data collection can be conducted over multiple sessions with a trial participant if required. Data will be digitised and entered onto our REDCap data management system managed by the CTU.

#### Plans to promote participant retention and complete follow-up

The participants will receive extensive information about the trial set-up and requirements during recruitment. We will attempt to collect follow-up data from participants who discontinue from the intervention.

#### Data management

The trial electronic database will be managed and operated as required by Good Clinical Practice. The site investigator or delegate will record all trial data within our electronic database (REDCap) provided by CTU. A record of patients who were screened as ineligible and those who were eligible and invited to take part but did not consent will be kept. The PIs are responsible for keeping a list of all consented patients, via the enrolment log. The investigator will ensure accuracy, completeness and timeliness of the data entered into the database.

Data will be checked according to a trial Data Management Plan and queries will be generated and sent to the site investigator for response using the REDCap database. Corrections resulting from these queries will be confirmed and sent back to STU. The queries and their responses will be stored in the audit trail of the electronic database.

Data will be analysed at the end of the trial once the database has been locked.

#### Confidentiality

All trial participant data will be pseudonymised and individual records identified using a unique participant identification number (PIN). A copy of the PIN will be kept securely within the site Investigator Site File (ISF). Minimal identifiable data to link participants' names and their PINs will be stored separately from the ISF.

The ISF containing original signed consent forms will be kept in secure premises. Access to the ISF will be restricted to researchers working on the trial. Sponsor representatives and auditors authorised to access the file.

### Statistical methods for primary and secondary outcomes

#### Quantitative outcomes

The primary outcome will be assessed using an intention-to-treat analysis. Quantitative outcome measures will be analysed by analysis of covariance, adjusting for baseline values of the outcome and any variables used in the randomisation process. Secondary outcomes will be analysed using linear or logistic regression models as appropriate reflecting the distribution of the outcome variable. The treatment effect will be estimated as an adjusted difference between sample means, presented with 95% CIs and p values. A statistical and health economic analysis plan (SHEAP) will be produced and finalised before data lock.

#### Process evaluation

A process evaluation will follow UK Medical Research Council guidelines^62^ and will include:

- Completion of a Template for Intervention Description and Replication proforma to specify the intervention.^63^
- A description of the procedures used as TAU.
- Analysis of the fidelity of treatment delivery (as described above) and adherence.
- Recording of inadvertent unblinding, trial-related adverse events and reasons for drop-out.
- Interview transcripts of patients’, therapists’ and carers’ views of the acceptability and efficacy of treatment, which will be subjected to a Thematic Analysis^64^ and to Framework Analysis^65^ for comparison.

#### Health economic outcomes

The health economic analysis will consist of a within-trial cost-effectiveness using cost–utility analysis of PES+EMDR versus TAU, assessed from the perspective of the UK NHS and personal social services (PSS) at 14 months follow-up.

Resource use and related costs of developing and delivering the intervention, including therapist training, will be recorded and measured. Due to the variability in the delivery of TAU across sites and participants, a bespoke questionnaire will be completed by research assistants to collect any psychological, psychologically informed or other treatment provided by members of the Learning Disability Team for participants allocated to TAU from patient records.

The costs (NHS, other health providers, social care) of supporting participants through the period of treatment will be collected using the Client Service Receipt Inventory–CSRI: ID version^66 67^ adapted for this trial. Resource use will be valued using the most up to date reference costs; otherwise, estimates from the literature, adjusted for inflation, will be used, as is standard for this tool. The CSRI-ID will be administered at baseline and 14-month follow-up.

Descriptive statistics will be used to summarise resource use, costs and outcomes by study arm. Incremental costs and quality-adjusted life-years (QALYs) will be analysed using appropriate regression methodology adjusting for stratification and minimisation variables. The net benefit framework will be used to assess the cost-effectiveness over a range of values for the QALY.

#### Interim and subgroup analyses

No interim analyses are planned. A series of subgroup analyses may be undertaken to determine whether PES+EMDR is more effective in certain participants. This may include completers vs non-completers and PTSD vs complex PTSD.

A complier average causal effect analysis will be considered should the data availability be sufficient to estimate the impact of non-compliance. Compliance will be defined as completing at least 80% of PES/EMDR sessions. A series of subgroup analyses on the primary outcomes will be considered considering differences in the IES-ID scores at 60 weeks for: (a) differing levels of general intellectual functioning, and (b) PTSD versus complex PTSD.

#### Methods in analysis to handle protocol non-adherence and any statistical methods to handle missing data

Every attempt will be made to minimise missing data, including follow-up of participants who discontinue treatment. Patterns and levels of missing data will be assessed. Procedures will be in place for validating all data and imputation will be considered if required.

#### Plans to give access to the full protocol, participant level data and statistical code

In line with the NIHR Open Access policy, following trial publication in a peer reviewed journal, a pseudonymised research dataset will be made publicly available by depositing in an on-line open access data repository.

### Oversight and monitoring

#### Composition of the coordinating centre and oversight committees

The Trial Management Group meets monthly and comprises the chief investigator, sponsor representative, the trial manager and other CTU staff.

The Site Management Group meets fortnightly and comprises the chief investigator and coinvestigators and the trial manager and other CTU staff as well as all the research assistants and PIs.

The TSC comprises a chair, statistician, representative of a national charity, an EMDR expert, the coinvestigator who oversees PPI, the coordinator of a service-user PPI group, and a carer.

The DMEC, which is independent of the sponsor and competing interests, comprises a statistician (in the chair), a senior clinical psychologist and a representative of a national ID charity.

All members of both the TSC and DMEC have signed a relevant charter and agreed Terms of Reference.

### Adverse event reporting and harms

In this trial, standard definitions of adverse events (untoward clinical occurrence experienced by a trial participant, which does not necessarily have a causal relationship with the intervention) and serious adverse events (results in death; is life threatening; requires hospitalisation or prolongation of an existing hospitalisation; results in persistent or significant disability or incapacity; or is otherwise considered medically significant by the chief investigator (CI) or site PI) are used.

The CI, the site PI or a delegate authorised in the site delegation log will assess each adverse event (AE) and serious AE (SAE) for seriousness, causality and expectedness. We will record and report only AEs/SAEs assessed as serious, unexpected and definitely, probably or possibly related to the intervention and the participant’s (patient or carer) involvement in the trial. In order to make this judgement, all AEs/SAEs will be recorded in the REDCap electronic trial database, and reviewed for expectedness by the CI.

Within 24 hours of receiving notification of an AE/SAE occurring, following consent and up to 4 weeks after the end of the intervention, the site PI or delegate will complete a trial AE/SAE form; assess the event’s seriousness and causality; specify actions taken, including any follow-up required. The Trial Manager will notify the CI of the event. The trial manager or CI may request data clarification from the site as necessary. A differing review by the CI will not result in a downgraded event.

If either the CI or PI assesses the AE/SAE as related and unexpected, the CI will notify the Research Ethics Committee (REC) that approved the trial, and the sponsor, within 24 hours of becoming aware of the event, even if that assessment is still provisional. The trial manager will report blinded cumulated AEs/SAEs to each meeting of the TSC. The Data Monitoring Committee (DMC) will receive unblinded reports and review all events. Site PIs will be notified of all such events. All emergency unblinding of related SAEs will be at the discretion of the PI or CI and will occur when required to ensure participant safety.

All unblinding events must be automatically notified by email to the trial office. The trial office will notify the REC, local R&D offices and the DMC. Details of the unblinding must be documented using an unblinding log and stored in a separate section of the ISF retained by the local clinical PI and the Trial Master File.

### Frequency and plans for auditing trial conduct

This trial may be subject to inspection and audit by Birmingham Community Healthcare NHS Foundation Trust under their remit as sponsor to ensure adherence to Good Clinical Practice and the UK Policy Framework for Health and Social Care Research. Site Investigators must make all trial documentation and related records available should any sponsor investigation be undertaken.

The sponsor has delegated central monitoring to the STU team. The following checks would be typical:

- Written informed consent has been documented appropriately.
- Screening and enrolment logs are complete.
- Data collected are consistent with protocol adherence.
- Case report forms (CRFs) are completed by authorised persons.
- SAE recording and reporting procedures are followed correctly.
- No key data are missing.
- Data are valid and accurate.
- Visits are within the protocol specified window.
- Delegation and training logs are complete and compliant.
- Review of recruitment rate, withdrawals and loss to follow-up.

Risk-based monitoring will be employed, using triggering techniques that enable resources to be focused on high-priority sites without compromising safety or quality of research.

Risk-based monitoring promotes the use of data to initiate a site visit only when justified by on-site workload or other quality triggers. The method involves the identification of risks and then links each risk with appropriate triggers that will initiate on-site or remote source data verification. Study risks may include:

- Past site performance.
- The number of participants and rate of site recruitment.
- Staff feedback on protocol compliance.
- Site contact.
- Record keeping.
- Information received from data management, such as missing CRFs, query rates and CRF completion delays.
- Inaccurate or repetitive data; and safety issues.

### Plans for communicating important protocol amendments to relevant parties (eg, trial participants, ethical committees)

All protocol amendments will be approved by the Sponsor and the REC. They will be communicated as appropriate to other interested parties (approved R&D departments, investigators, research staff, clinical PIs, oversight committees, PPI groups) by the trial manager.

### Dissemination plans

Ownership of the data arising from this research project resides with the research project team and their respective employers and the sponsor. On completion of the research project, the research project data will be analysed, and a final research project report will be prepared which will be peer reviewed and published. Two public dissemination meetings will be held, and the findings will additionally be reported at relevant national and international scientific meetings. The International Committee of Medical Journal Editors guidance will be used to determine authorship. Professional writers will not be used.

In line with the NIHR Open Access policy following trial publication in a peer-reviewed journal, the non-person identifiable research dataset will be made publicly available by depositing in an on-line open access data repository.

### Discussion

#### Design issues

The design of this trial involved a number of significant decisions which included: (a) using ICD-11 diagnostic criteria for both PTSD and complex PTSD as opposed to DSM-5, (b) recruiting psychologists experienced in working with adults with intellectual disabilities and training them to deliver EMDR as opposed to recruiting EMDR therapists and training them to work with adults with intellectual disabilities, (c) the incorporation of extended PES phase before commencing trauma confrontation, (d) a loosening of the ICD-11 diagnostic criteria for PTSD for use with people with intellectual disabilities based on our experiences of completing a previous feasibility study^44^ and evidence to indicate that people with intellectual disabilities may present with atypical trauma symptoms,^68^ (e) designing trial procedures so that patients had to recount their trauma history once and (f) avoiding completing a full assessment of general intellectual functioning and adaptive behaviour for those with an existing diagnosis of an intellectual disability. Instead, all trial participants completed the Weschler Abbreviated Intelligence Scale–II.^69^ Finally, we also decided that TAU should not involve another trauma-focused psychological therapy such as TF-CBT.

#### Impact of the COVID-19 pandemic

As with many projects, the trial was severely impacted by the COVID-19 pandemic. All non-COVID research was stopped across our participating NHS sites, and it was well into 2022 and early 2023 before full permission to resume the trial at individual sites was restored. However, the pandemic left a legacy of waiting lists and a shortage of staff, with significant stress and burnout in existing staff. Our schedule of fortnightly site meetings was maintained throughout the pandemic and its aftermath, in order to maintain morale among the investigators and research staff, and to maintain therapist engagement and motivation by inviting attendance of the senior clinician in each Trust. It remains the case that staff remain focused on their clinical waiting lists and are not prioritising research.

A number of adjustments were made to cope with the impact of the pandemic, including training of extra cohorts of therapists, which was originally conceived as a contingency measure but became a necessity. We originally required therapists to fully complete a ‘training case’ before delivering EMDR as a trial therapist, but we loosened this requirement to having worked with a training case for at least 1 month prior to delivering EMDR as a therapist. We also set about recruiting additional NHS sites with psychologists specialising in working with people with intellectual disabilities who were already trained and experienced EMDR therapists.

#### Limitations

A significant limitation of this trial is that it may be difficult to recruit participants from ethnic minority communities. One reason is that we considered it impractical to include participants who require the support of an interpreter. A further limitation is that the trial does not address the political dimension of clients being traumatised by poor quality services and living environments. This issue has been highlighted by a succession of scandals involving mistreatment of people with intellectual disabilities.^70 71^ The trial does not include an analysis of the quality of trial participant social and physical environments, which are likely to impact significantly on treatment outcomes.

### Trial status

This paper is based on v.4.0 of the trial protocol, 1 February 2024. Recruitment commenced in March 2022 and ceased on 31 December 2024. Participants are currently being followed up, and the trial is expected to complete in July 2026. Trial registration details can be found in online supplemental material: appendix 4.

### Ethical opinion

A favourable ethical opinion was gained for this trial from the Wales NHS REC 3 on 31 Jul 2019 (Ref: 19/WA/0173).

## Supplementary material

10.1136/bmjopen-2025-108818 online supplemental file 1
