# Supplementary material for: A multicentred two-arm parallel single-blind superiority randomised controlled trial comparing psychological and emotional stabilisation with eye movement desensitisation and reprocessing and treatment-as-usual to treatment-as-usual with adults with intellectual disabilities who have post-traumatic stress disorder (the Trauma-AID trial): protocol
Source: BMJ Open. 2025 Dec 17;15(12):e108818. doi: 10.1136/bmjopen-2025-108818 (PMC12716544; doi:10.1136/bmjopen-2025-108818)
Supplement: online supplemental file 1 [file bmjopen-15-12-s001.docx]

**Supplemental Material**

**Appendix 1: Participating NHS Trusts**

Birmingham Community Healthcare NHS Foundation Trust

Black Country Healthcare NHS Foundation Trust

Coventry and Warwickshire Partnership NHS Trust

Hertfordshire Partnership University NHS Foundation Trust

Kent and Medway Social Care Partnership NHS Trust

Leicester Partnership NHS Trust

Livewell Southwest

Midlands Partnership University NHS Foundation Trust

Norfolk Community Health and Care NHS Trust

Oxford Health NHS Foundation Trust

Oxleas NHS Foundation Trust

Sussex Partnership NHS Foundation Trust

**Appendix 2: Remote consent procedure**

If remote consent is required, then the following procedure is followed:

- The therapist will ascertain from the patient if there is preference or need to work remotely.
- The patient is introduced to the trial by the therapist reading the Information Script during the course of a scheduled online meeting
- If the patient expresses interest in participating, the therapist can pass relevant contact information (service user’s mobile/telephone/email) to the research assistant (RA).
- The RA immediately mails out copies of the PIS and Informed Consent Form (ICF) (and easy read summaries) and confirms this to the therapist.
- At the next session, the therapist goes through the PIS with the patient, inviting questions and checking understanding. The patient is encouraged to have another person present, for example a carer.
- The therapist contacts the patient by phone or video and ascertains interest in the trial. If the patient expresses interest the therapist passes relevant contact information (service user’s mobile/telephone/email) to the RA, as well as any recommendations about how best to build rapport with the service user.
- The RA contacts the participant by phone or video and introduces the trial by using the Information Script.
- If the patient is still interested in participating, the RA arranges a remote video or telephone appointment at least 2 days after the letter should arrive, and immediately mails or emails a confirmation along with copies of the PIS, easy-read summary and ICF.
- At the appointment, the RA goes through the PIS with the patient, inviting questions and checking understanding. The patient is encouraged to have another person present, for example a carer.
- The person taking consent goes through the ICF with the patient and either records a video of the participant signing it, or takes a photograph of the screen, ensuring that the recording or photograph includes a legible record of the signed form.
- If the ICF has been video-recorded the recording is saved locally, where a member of the research team prints out the ICF, signs it, and records the name of the person who took consent.
- If the ICF has been photographed, the person taking consent prints out the signed form, adds their own signature, and files the form locally.
- The signed form is securely filed, and the video of signing is securely stored on an access-controlled drive.
- A narrative account of the process along with relevant documents and files are added to the local clinical note system.

**Appendix 3: Participant Information Sheets and Consent Forms**

Participant Information Sheet - **Service User-MainV4_01Feb2024**

| **IRAS ID:**  **Trial Name:** | **260514**  **Eye movement desensitisation and reprocessing for symptoms of post-traumatic stress disorder in adults with intellectual disabilities (Trauma-AID)** |
| --- | --- |
|  | |

**Introduction**

We would like to invite you to participate in a research trial. Before you decide whether or not to take part, it is important for you to understand why we are doing this research and what it involves. Please take time to read this sheet carefully. Please talk to your family and friends about the trial if you wish. You can ask us if anything is unclear and a member of our research team will answer any questions you may have.

**Why are we doing this research study?**

Sometimes people with learning disabilities are badly treated, or have bad things happen to them. This can mean that they sometimes suffer from a condition called post-traumatic stress disorder (shortened to PTSD). People with PTSD are often frightened that bad things might happen again, and it can stop them from going out and enjoying life to the full.

Psychologists and therapists are people who are trained to look at how you feel and to help you feel better. They have some ways of helping people with PTSD. We have completed a small study and now will be looking more at a treatment that works quite well for some people with PTSD. This treatment is known as Eye Movement Desensitization and Reprocessing (shortened to EMDR). EMDR is in two parts. In the first part, people work with a psychologist or therapist to learn ways of coping with bad memories that they find upsetting. In the second part, the psychologist or therapist helps them to remember those bad things without the memory being as upsetting as it usually is. To do this, the psychologist or therapist asks people about bad things that have happened to them while they also do something else such as moving their eyes. We will also give you a pictorial leaflet that tells you more about EMDR.

In this study, we want to see if EMDR is better than other treatments for helping people to remember bad things without them feeling as upset as they usually do.

**Why have I been invited?**

You have been invited to take part in the study because you have a learning disability and have been referred for treatment of PTSD to a team that is taking part in this study.

**Do I have to take part?**

No. It is up to you to decide whether or not you want to take part in this study. If you decide to take part, you are free to stop at any time, without giving a reason. If you decide not to take part, or later to leave, this will not affect your care in any way.

**What if I do not want to take part?**

If you do not wish to take part, that is perfectly OK. Nothing will happen as a result. Your treatment in the NHS will continue exactly as it would if we had not approached you.

**When do I have to decide whether or not I will take part?**

You have at least three days to decide if you wish to take part. The person who gave you this information sheet will talk to you again and answer any questions you might have.

**What will happen if I decide to take part?**

Because of the COVID illness that is going around, we might not be able to meet with people face-to-face. If we can’t meet face to face, we will send you a link to use a video meeting application, like Zoom or Teams, to meet with a therapist or researcher. In some cases, we contact you by telephone.

If you agree to take part, we will first ask you to sign a consent form to say that we have talked to you about the study and that you understand what it is about and what will happen to you during the study. The consent form shows that you agree to take part and that we can use the information we collect about you. We may record some or all of the consent conversation to show that you understand what we are doing. We will also make a record of you signing of the consent form too.

Once you have given your consent, we will ask you to complete three questionnaires and some tests. This will take about an hour. With your permission we will also ask your carer some questions about you. This will help us to see if you are eligible to take part the trial

If you are not eligible to take part in this trial, your psychologist or therapist will talk to you about what would be the best treatment for you.

If you are eligible, we will ask you to do some more questionnaires, which will take about an hour. We would also like to ask your carer some more questions about you. We will then select your treatment plan by chance (randomly – like tossing a coin).

You will either have EMDR or the usual treatment that the psychologist or therapist would usually give someone (known as standard care). The treatment is being chosen this way because we don’t know which of the two treatments works better.

We will ask you and your carer to complete the same questionnaires again four months later (half way through your treatment) and again after eight months (when your treatment should have finished). Then we would like to ask you and your carer to complete the questionnaires six months later, to see if you are still feeling like you did at the end of the treatment.

So if you are eligible for the trial, altogether, we will ask you and your carer to complete the questionnaires four times: before the start of your treatment, after 4 months of treatment, after 8 months of treatment, and another 6 months later. Each time we do this it will take about an hour.

There are two other things that we might ask you to do. We only need a few people to do these things, so they might not happen.

- We might ask if you would mind us making a recording of one or two of your sessions with your psychologist or therapist. This is so that we can check that the person treating you is doing things in the way we want for the trial. (We are not checking on you.) The person who listens to the recording to check this will be far away in another part of the country, and would not know who you are or who your therapist is.
- We might ask if you would mind talking to a researcher after your treatment has finished. We would ask what you think about the treatment you had and if you have ideas about how we could do it better. We would ask your permission to record the conversation.

**What will you do with the information I provide?**

We will use the information to find out if EMDR works better than “treatment as usual”. After the study has finished, we plan to publish the results in scientific journals and present them at medical conferences. We will also send a report of all the results to the people who gave us the money to do the research – the National Institute for Health Research.

You will not be identified in any of the articles or talks. We might use exact words from our conversation with you (known as a quotation) to help us explain the study but no-one will know the words came from you.

**What are the possible benefits of taking part?**

You may or may not benefit from the treatment you receive. You will have the opportunity to talk about things that have happened to you in the past and you may find it useful to have someone to talk to about what happened and how you feel. You will also help us to know if EMDR does help people with learning disabilities and PTSD, and this could benefit people with learning disabilities in the future

**What are the possible risks of taking part?**

We will be asking you about bad things that have happened to you, and you might find some of the questions or memories upsetting. These are the same things that you will be discussing with the therapist treating you, and they will support you through any difficult questions or feelings that you have. You will be free to take a break or stop completely at any time.

**How have patients and the public been involved in this study?**

Members of the public have been involved in discussions and helped develop this research project.

**Who are the researchers?**

The people running this study are (i) clinical psychologists from NHS Trusts in the west midlands and the London area, and (ii) other experienced researchers based in several UK universities.

The study is managed (known as sponsored) by Birmingham Community Healthcare NHS Foundation Trust Unit 3 Priestley Wharf, 20 Holt Street, Aston, Birmingham, B7 4BN; Telephone: 0121 466 7078; Email:research.innovation@bhamcommunity.nhs.uk.

The person in charge of looking after the study, known as the Chief Investigator is Professor Peter Langdon. If you want to ask him anything, please contact the manager of this research study who will pass on your question Stephen Hiles, telephone 01792602624, email trauma-aid@swansea.ac.uk.

**What if I have a question or would like more information?**

The person in charge in this Trust is (local PI details). If you have a question or would like more information, please contact him/her, or contact the Trial Manager of this research study using the information above.

If they cannot answer your question themselves, they will know someone else in the research team who can and make sure someone speaks to you and answers your questions.

**IF YOU THINK YOU MAY BE INTERESTED IN BEING PART OF THIS RESEARCH, PLEASE KEEP ON READING.**

**Will any information I provide be confidential?**

Yes. All information collected from you will be kept in confidence, as far as possible. We will not write or say anything that would identify you, unless you tell us in writing that it would be alright to identify you. This means no one will ever know from anything that we have written or said that you had been part of this study unless you have said that it is OK. However, if you tell the researchers something they think is likely to result in immediate harm to yourself or others, they may have to disclose this in accordance with the law and local safeguarding procedures. We will always talk to you first if this was something we were thinking of doing.

**What will happen to the information that you collect from me?**

The trial researchers will only use information that you have agreed we can collect from you.

Your questionnaire answers and conversations will be looked at along with the answers and conversations collected from other patients in the study.

The law tells us that we must let you know that we collect research information in the public interest. The Birmingham Community Healthcare NHS Foundation Trust, as sponsor, is known as the data controller and will make sure that we look after your information by using it properly and keeping it safe.

The researchers will use your name and contact details to get in touch with you about your trial visits. Your contact details will always be stored separately from the recordings and questionnaire that we have for you. Any recordings and questionnaires that we collect from you will only have a code number that we give you. This will make sure that everything you give us remains confidential. We will keep your contact details for 6 – 12 months after the trial has finished so that we can let you know about the results of the study.

If we record any conversation this might be given to a company who specialise in typing up what you said (known as a transcript). Names, places or any other information mentioned in the conversation that could help to identify you will be removed from the transcript. The recordings would be sent from the researchers to the company and back safely and securely, to ensure they remain confidential. With your permission, we will store the typed conversation, your questionnaires and consent forms securely at Swansea Trials Unit for 5 years after the study has finished. We may use the information collected for future analysis if we have any further questions.

All conversations will be typed up at the earliest opportunity. Once the transcripts are checked to show they are accurate, the recordings will be destroyed.

Your rights to access, change or move your personal information may be limited, as we need to manage your information in particular ways in order for us to show that the research is reliable and accurate. Further information about your rights and your personal data is available at: <http://www.bhamcommunity.nhs.uk/about-us/corporate-information/research-and-innovation/>

Information collected during the study may be looked at by people whose job is to check that we are doing the research properly. These include approved individuals from the Sponsor, Swansea University, the NHS Trust or the National Institute of Health Research. They would only look at information that is important to your participation in this research.

**What will happen if I don’t want to carry on with the study?**

Your participation in this trial is up to you. You may withdraw your consent at any time without the need to give us a reason. If you decide to withdraw, all questionnaires completed would be destroyed, as would any recordings and typed information of therapy sessions or conversations. However, it will not be possible to withdraw data that does not identify you from the study once the analysis is complete or the findings from the study are published. None of this would in any way affect the standard of care you receive during your treatment.

**Will I be paid for taking part in the study?**

Taking part in our trial will take up some of your time. We will make every effort to make sure that the inconvenience to you is small. We will also give you a voucher worth £10 for each of the 5 sessions where you are completing our questionnaires which you will be able to spend in high street shops.

Our researcher will come to you (for example, to your home or where your therapy happens), so taking part in the study should not cost you anything in travel expenses. But if you did have to make a special journey to meet us, we would reimburse the cost.

**Who is funding the research and who has reviewed it?**

The people who gave us the money to do the study are called the National Institute for Health Research.

An independent group of people, called a Research Ethics Committee, looks at research studies. The committee look to protect the safety, rights, wellbeing and dignity of people who take part in research studies. This study has been reviewed and given a favourable opinion by Wales REC 3. The study has also been given permission from your hospital’s Research and Development Office.

**What if there is a problem?**

Given the kind of trial, it is not likely that you will suffer harm by taking part. However, the Birmingham Community Healthcare NHS Foundation Trust, as sponsor, has appropriate insurance in place in the unlikely event that you suffer any harm because you took part in this trial.

If you wish to complain about how anyone talked to you about the trial or treated you while you took part, you can speak to the researchers who you see to complete the questionnaires. You will be given you a card with their contact details. If you have a problem, we would like it if you could talk to the researcher(s) in the first instance if you are able to.

If you are not happy with the answers from the researchers, or you want to talk to someone who is not part of the research team then please contact the Research and Innovation Department by emailing [research.innovation@bhamcommunity.nhs.uk](mailto:research.innovation@bhamcommunity.nhs.uk) or telephone: 0121 466 7078.

Should you be harmed in any way during the research you may have grounds for a legal action for compensation against the NHS Trust, but you may have to pay your legal costs to do this. The normal NHS complaints mechanisms will still be available to you.

You can also contact the local Customer Services Department/Patient Liaison Service (PALS) for advice or support. Their contact details are:

Email: [contact.bchc@nhs.net](mailto:contact.bchc@nhs.net) or [complaints.bchc@nhs.net](mailto:complaints.bchc@nhs.net)

Free phone: 0800 9172855

Website: <http://www.bhamcommunity.nhs.uk/contact>

**Thank You**

Thank you for thinking about taking part in this research study and reading this information sheet, which is yours to keep.

Easy Read Participant Information Sheet**: Info Summary-Main V2 07 Oct 2021**

| We want to find out how to help people who have had bad things happen to them | | | | IMAGE REDACTED DUE TO JOURNAL POLICY | |
| --- | --- | --- | --- | --- | --- |
| I will do two lots of questionnaires with you | | | | IMAGE REDACTED DUE TO JOURNAL POLICY | |
| That will take about one hour each time | | | | IMAGE REDACTED DUE TO JOURNAL POLICY | |
|  | | | |  | |
| I will also ask your carer some questions | | | | IMAGE REDACTED DUE TO JOURNAL POLICY | |
| You will get help from a psychologist | | IMAGE REDACTED DUE TO JOURNAL POLICY | | | |
| We don’t know yet what sort of help you will get.  Some of your treatment might be over video or the phone | | IMAGE REDACTED DUE TO JOURNAL POLICY | | | |
| We might want to record one or two sessions | IMAGE REDACTED DUE TO JOURNAL POLICY | | | | |
| I will come back and see you after a few months | IMAGE REDACTED DUE TO JOURNAL POLICY | | | | |
| I will ask you the same questions as before  We will do this twice more | IMAGE REDACTED DUE TO JOURNAL POLICY | | | | |
| I might ask you what you think about the therapy you did | | IMAGE REDACTED DUE TO JOURNAL POLICY | | | |
| We will pay you for answering our questions | | IMAGE REDACTED DUE TO JOURNAL POLICY | | | |
| We will not talk about you to anyone else without asking you first.   - You can say ‘Yes’ - You can say ‘No’ | | | IMAGE REDACTED DUE TO JOURNAL POLICY | |  |
| It’s your decision to take part or not.  - You can say ‘Yes’  - You can say ‘No’ | | | IMAGE REDACTED DUE TO JOURNAL POLICY | |  |
| If you want to ask any questions you can phone me.  You have my phone number.  Your carer will help you. | | | IMAGE REDACTED DUE TO JOURNAL POLICY | |  |
| If you need to complain about anything, please talk to your carer. | | | IMAGE REDACTED DUE TO JOURNAL POLICY | |  |

Consent Form: **ICF Service User Main easy read V1 07 Oct 2021**

| **Trial Name:** | **Eye movement desensitisation and reprocessing for symptoms of post-traumatic stress disorder in adults with intellectual disabilities (Trauma-AID)** | | |
| --- | --- | --- | --- |
| **Chief Investigator:** | **Professor Peter Langdon** | | |
| **Principal Investigator:** |  | | |
| **Participant Identification number:** | |  |  |

|  |  | **Please initial each box** |
| --- | --- | --- |
| IMAGE REDACTED DUE TO JOURNAL POLICY | I have read the information sheet, or had it read to me, Info Summary-Main V2 07 Oct 2021 for the above study. I understand what it tells me. |  |
| IMAGE REDACTED DUE TO JOURNAL POLICY | I have had time to think about it and talk about joining in with friends, family or carers.  I have been able to ask questions about the study. All my questions have been answered. |  |
| IMAGE REDACTED DUE TO JOURNAL POLICY | I know that I do not have to take part in this study.  I know that I can stop taking part in this study whenever I want to. I do not need to give a reason to leave the study.  If I leave the study, my medical care or legal rights will not be affected |  |
| IMAGE REDACTED DUE TO JOURNAL POLICY | I agree to the random selection of my treatment. This means that I might get EMDR or might get another treatment. |  |
| IMAGE REDACTED DUE TO JOURNAL POLICY | I understand that some of my treatment might be over video or the phone. |  |
| IMAGE REDACTED DUE TO JOURNAL POLICY | I agree to try and do the questionnaires with a researcher. I understand that I can take a break or stop completing them if I wish. |  |
| IMAGE REDACTED DUE TO JOURNAL POLICY | I understand that:   - one or two of my therapy sessions might be recorded - I might be asked to talk about treatment that I have had. - conversations with me about my treatment can be recorded and written out in full (transcribed) - any recordings may be transcribed by the research team or by a specialist company. - exact words from my recording (a quotation) may be used in papers, reports or conferences to help explain the study, but no one would know they came from me. My name will not be used. |  |
| IMAGE REDACTED DUE TO JOURNAL POLICY | I understand that my study records, questionnaires or transcripts of my recordings may be looked at by people from the Trauma-AID research team or the [local NHS Trust]. I give permission for these individuals to have access to my trial records and all information collected.  I understand that the research team might look at my health records to collect information about me. |  |
| IMAGE REDACTED DUE TO JOURNAL POLICY | I agree that my carer, friend or relative may be asked to complete questionnaires about me and to talk about my treatment. |  |
| IMAGE REDACTED DUE TO JOURNAL POLICY | I understand that information collected will be kept confidential. However, if the researchers feel that I or others are at risk of harm, they may have to talk to me about telling someone else. This in line with the law and local safeguarding procedures and is to make sure everyone is safe. |  |
| IMAGE REDACTED DUE TO JOURNAL POLICY | I agree to take part in the above study. |  |

| **Your name** | **Date** | **Signature** |
| --- | --- | --- |
|  |  |  |

| **Name of person taking consent (in capitals)** | **Date** | **Signature** |
| --- | --- | --- |
|  |  |  |

| **Name of witness (in capitals)** | **Date** | **Signature** |
| --- | --- | --- |
|  |  |  |

Carer Information Sheet: **PIS carer-main V4 01 Feb 2024**

| **IRAS ID:**  **Trial Name:** | **260514**  **Eye movement desensitisation and reprocessing for symptoms of post-traumatic stress disorder in adults with intellectual disabilities (Trauma-AID)** |
| --- | --- |
|  | |

**Introduction**

We would like to invite you to participate in a research trial. Before you decide whether or not to take part, it is important for you to understand why we are doing this research and what it involves. Please take time to read this sheet carefully. Please talk to your family and friends about the trial if you wish. You can ask us if anything is unclear and a member of our research team will answer any questions you may have.

**Why are we doing this research study?**

Sometimes people with learning disabilities are badly treated, or have bad things happen to them. This can mean that they sometimes suffer from a condition called post-traumatic stress disorder (often shortened to PTSD). People with PTSD are often frightened that bad things might happen again, and it can stop them from going out and enjoying life to the full.

Psychologists and therapists are people who are trained to look at how people feel and to help them feel better. They have some ways of helping people with PTSD. We have completed a small study and now will be looking more at that a treatment that works quite well for some people with PTSD. This treatment is known as Eye Movement Desensitization and Reprocessing (shortened to EMDR). EMDR is in two parts. In the first part, people work with a psychologist or therapist to learn ways of coping with bad memories that they find upsetting. In the second part, the therapist helps them to remember those bad things without the memory being as upsetting as it usually is. This involves the psychologist or therapist asking people about bad things that have happened to them while they also do something else such as moving their eyes. We will also give you a pictorial leaflet that tells you more about EMDR.

In this study, we want to see if EMDR is better than other treatments for helping people to remember bad things without them feeling as upset as they usually do.

**Why have I been invited?**

You have been invited to take part in the study because you are the carer of a person who has a learning disability and has recently been referred for treatment of PTSD to a team that is taking part in this study. We would like to ask you some questions about the person you care for. The person you care for has given us permission to talk to you about them.

**Do I have to take part?**

No. It is up to you to decide whether or not you want to take part in this study. If you decide to take part, you are free to stop at any time, without giving a reason. If you decide not to take part, or later to leave, this will not affect in any way the care that the person you care for receives.

**What if I do not want to take part?**

If you do not wish to take part, that is perfectly OK. Nothing will happen as a result.

**When do I have to decide whether or not I will take part?**

You have at least three days to decide if you wish to take part. The person who gave you this information sheet will talk to you again and answer any questions you might have.

**What will happen if I decide to take part?**

Because of the COVID illness that is going around, we might not be able to meet with people face-to-face. If we can’t meet face to face, we will send you a link to use a video meeting application, like Zoom or Teams, to meet with a therapist or researcher. In some cases, we contact you by telephone.

If you agree to take part, we will first ask you to sign a consent form to say that we have talked to you about the study and that you understand what it is about and what will happen to you during the study. The consent form shows that you agree to take part and that we can use the information we collect about you. We will record some or all of the consent conversation to show that you understand what we are doing. We will also make a record of you signing of the consent form too.

Once you have given consent, we will ask you to complete a questionnaire to see if the person you care for is eligible to take part the trial. This will take about 20 minutes.

If it turns out that the person you care for is eligible, we will ask you to do some more questionnaires, which will take less than an hour. We will then select the treatment plan by chance (randomly – like tossing a coin). The person you care for will either have EMDR or the usual treatment that the psychologist or therapist would usually give someone (known as standard care). The treatment is being chosen this way because we don’t know which of the two treatments works better.

We will ask you to complete the same questionnaires again four months later (half way through the treatment) and again after eight months (when the treatment should have finished). Then we would like to ask you to complete the questionnaires six months later, to see if the person you care for is still feeling like they did at the end of the treatment.

Altogether, we will ask you to complete the questionnaires four times: before the start of your treatment, after 4 months of treatment, after 8 months of treatment, and another 6 months later. Each time we do this it will take about an hour.

The therapist might ask you to help with aspects of the therapy. This would be between you, the person you care for and the therapist. It is not part of the research study. However, if you are asked to help in this way, we might ask if you would mind talking to a researcher after the treatment has finished. We would ask you your views of the treatment and if you have ideas about how we could do it better. This would take about 20 minutes. We would ask your permission to record the conversation. We only need a few people to do this, so you might not be asked.

**What will you do with the information I provide?**

We will use the information to find out if EMDR works better than “treatment as usual”. We will write articles and give talks about the research, so that other therapists know about the treatment and could use it with their clients if it works.

You will not be identified in any of the articles or talks. We might use exact words from our conversation with you (known as a quotation) to help us explain the study but no-one will know the words came from you.

**What are the possible benefits of taking part?**

There are no direct benefits to you or the person you care for from taking part. However, you will help us to know if EMDR does help people with learning disabilities and PTSD.

**What are the possible risks of taking part?**

We will be asking you questions about the person you care for, and perhaps about what you think of the therapy the person received. We are not aware of any risks. You will be free to take a break or stop completely at any time.

**How have patients and the public been involved in this study?**

Members of the public have been involved in discussions and helped develop this research project.

**Who are the researchers?**

The people running this study are (i) clinical psychologists from NHS Trusts in the west midlands and the London area, and (ii) other experienced researchers based in several UK universities.

The study is managed (known as sponsored) by Birmingham Community Healthcare NHS Foundation Trust Unit 3 Priestley Wharf, 20 Holt Street, Aston, Birmingham, B7 4BN; Telephone: 0121 466 7078; Email: research.innovation@bhamcommunity.nhs.uk.

The person in charge of looking after the study, known as the Chief Investigator is Professor Peter Langdon. If you want to ask him anything, please contact the manager of this research study who will pass on your question Stephen Hiles, telephone 01792602624, email trauma-aid@swansea.ac.uk

**What if I have a question or would like more information?**

The person in charge in this Trust is (local PI details). If you have a question or would like more information, please contact him/her, or contact the Trial Manager of this research study using the information above.

If they cannot answer your question themselves, they will know someone else in the research team who can and make sure someone speaks to you and answers your questions.

**IF YOU THINK YOU MAY BE INTERESTED IN BEING PART OF THIS RESEARCH, PLEASE KEEP ON READING.**

**Will any information I provide be confidential?**

Yes. All information collected from you will be kept in confidence, as far as possible. We will not write or say anything that would identify you, unless you tell us in writing that it would be alright to identify you. This means no one will ever know from anything that we have written or said that you had been part of this study unless you have said that it is OK. However, if you tell the researchers something they think is likely to result in immediate harm to yourself or others, they may have to disclose this in accordance with the law and local safeguarding procedures. We will always talk to you first if this was something we were thinking of doing.

**What will happen to the information that you collect from me?**

The trial researchers will only use information that you have agreed we can collect from you.

Your questionnaire answers and conversations will be looked at along with the answers and conversations collected from other carers in the study.

The law tells us that we must let you know that we collect research information in the public interest. The Birmingham Community Healthcare NHS Foundation Trust, as sponsor, is known as the data controller and will make sure that we look after your information by using it properly and keeping it safe.

The researchers will use your name and contact details to get in touch with you about your trial visits. Your contact details will always be stored separately from the recordings and questionnaire that we have for you. Any recordings and questionnaires that we collect from you will only have a code number that we give you. This will make sure that everything you give us remains confidential. We will keep your contact details for 6 – 12 months after the trial has finished so that we can let you know about the results of the study.

If we record any conversation this might be given to a company who specialise in typing up what you said (known as a transcript). Names, places or any other information mentioned in the conversation that could help to identify you will be removed from the transcript. The recordings would be sent from the researchers to the company and back safely and securely, to ensure they remain confidential. With your permission, we will store the typed conversation, your questionnaires and consent forms securely at Swansea Trials Unit for 5 years after the study has finished. We may use the information collected for future analysis if we have any further questions.

All conversations will be typed up at the earliest opportunity. Once the transcripts are checked to show they are accurate, the recordings will be destroyed.

Your rights to access, change or move your personal information may be limited, as we need to manage your information in particular ways in order for us to show that the research is reliable and accurate. Further information about your rights and your personal data is available at: <http://www.bhamcommunity.nhs.uk/about-us/corporate-information/research-and-innovation/>.

Information collected during the study may be looked at by people whose job is to check that we are doing the research properly. These include approved individuals from the Sponsor, Swansea University, the NHS Trust or the National Institute of Health Research. They would only look at information that is important to your participation in this research.

**What will happen if I don’t want to carry on with the study?**

Your participation in this trial is up to you. You may withdraw your consent at any time without the need to give us a reason. If you decide to withdraw, you can ask for the completed questionnaires to be destroyed, and if a conversation has taken been recorded you can ask for recordings and typed information of the conversation to be destroyed. However, it will not be possible to withdraw data that does not identify you from the study once the analysis is complete or the findings from the study are published. None of this would in any way affect the standard of care that the person you care for receives during treatment.

**Will I be paid for taking part in the study?**

Taking part in our trial will take up some of your time. We will make every effort to make sure that the inconvenience to you is small. The researcher will come to you, so you will not incur any travel costs. If you are a paid carer, we will not pay for your time. But if you are not paid as a carer – that is, if you do your caring as an unpaid family member or friend – we will give you a voucher worth £10 for each of the 4 sessions where you are completing our questionnaires. We will give you that £10 voucher at each visit and you will be able to spend it in high street shops.

Our researcher will come to you (for example, to your home or place of work), so taking part in the study should not cost you anything in travel expenses. But if you did have to make a special journey to meet us, we would reimburse the cost.

**Who is funding the research and who has reviewed it?**

The people who gave us the money to do the study are called the National Institute for Health Research.

An independent group of people, called a Research Ethics Committee, looks at research studies. The committee look to protect the safety, rights, wellbeing and dignity of people who take part in research studies. This study has been reviewed and given a favourable opinion by Wales REC 3. The study has also been given permission from your hospital’s Research and Development Office.

**What if there is a problem?**

Given the kind of trial, it is most unlikely that you will suffer harm by taking part. However, the Birmingham Community Healthcare NHS Foundation Trust, as sponsor, has appropriate insurance in place in the unlikely event that you suffer any harm because you took part in this trial.

If you wish to complain about how anyone talked to you about the trial or treated you while you took part, you can speak to the researchers who you see to complete the questionnaires. You will be given you a card with their contact details. If you have a problem, we would like it if you could talk to the researcher(s) in the first instance if you are able to.

If you are not happy with the answers from the researchers, or you want to talk to someone who is not part of the research team then please contact the Research and Innovation Department by emailing [research.innovation@bhamcommunity.nhs.uk](mailto:research.innovation@bhamcommunity.nhs.uk) or telephone: 0121 466 7078.

Should you be harmed in any way during the research you may have grounds for a legal action for compensation against the NHS Trust, but you may have to pay your legal costs to do this. The normal NHS complaints mechanisms will still be available to you.

You can also contact the local Customer Services Department/Patient Liaison Service (PALS for advice or support. Their contact details are:

Email: [contact.bchc@nhs.net](mailto:contact.bchc@nhs.net) or [complaints.bchc@nhs.net](mailto:complaints.bchc@nhs.net)

Free phone: 0800 9172855

Website: <http://www.bhamcommunity.nhs.uk/contact>

**Thank You**

Thank you for thinking about taking part in this research study and reading this information sheet, which is yours to keep.

Carer Consent Form: ICF Carer Main V2 07 Oct 2021

| **Trial Name:** | **Eye movement desensitisation and reprocessing for symptoms of post-traumatic stress disorder in adults with intellectual disabilities (Trauma-AID)** |
| --- | --- |
| **Chief Investigator:** | **Professor Peter Langdon** |
| **Principal Investigator:** | **Local Details to be added** |

| **Carer Identification number:** |  | **This consent form relates to Participant number:** |  |
| --- | --- | --- | --- |

|  | **Please initial each box** |
| --- | --- |
| 1. I confirm that I have read and understand the information sheet [version XX dated dd.mm.yyyy] for the above study. |  |
| 1. I confirm that I have had enough time to consider whether or not I want to take part and that all my questions have been answered in full. |  |
| 1. I understand that my taking part is voluntary and that I am free to stop taking part at any time without giving any reason. |  |
| 1. I understand that I will be asked questions about the person who I help care for and that they have agreed to my doing this. |  |
| 1. I understand that we may switch from meeting face-to-face to meeting via a video link, or from meeting via a video link to meeting face-to-face |  |
| 1. I agree that conversations that I have about the person who I help care for may be audio- and video- recorded and will be transcribed. |  |
| 1. I understand that conversations may be transcribed by the research team or by a specialist company |  |
| 1. I understand that anonymous quotations from my conversation may be used in papers, reports or conferences to help explain the study. |  |
| 1. I agree to take part in the above study. |  |

| **Your name (in capitals)** | **Date** | **Signature** |
| --- | --- | --- |
|  |  |  |

| **Name of person taking consent (in capitals)** | **Date** | **Signature** |
| --- | --- | --- |
|  |  |  |

**Appendix 4: Trauma-AID: Trial Registration Data**

| Primary registry and trial identifying number | Trial ID: ISRCTN35167485  Link: <https://www.isrctn.com/ISRCTN35167485> |
| --- | --- |
| Date of Registration | 22 Jul 2019 |
| Secondary identifying numbers | CPMS 42218  NIHR 17/125/04  NHS Research Ethics Committee: 19/WA/0173 |
| Sources of monetary or material support | This research is funded by the UK National Institute for Health and Care Research (Ref: 17/125/04). The views expressed are those of the authors and not necessarily those of the NIHR or the Department of Health and Social Care. |
| Primary Sponsor | Birmingham Community Healthcare NHS Foundation Trust |
| Secondary Sponsor | Not Applicable |
| Contact for Public Queries | Professor Peter Langdon (P.Langdon@bham.ac.uk) |
| Contact for Scientific Enquires | Professor Peter Langdon (P.Langdon@bham.ac.uk) |
| Public Title | Eye movement desensitisation and reprocessing for symptoms of post-traumatic stress disorder in adults with intellectual disabilities (Trauma-AID) |
| Scientific Title | A multicentred two-arm parallel single-blind superiority randomised controlled trial comparing psychological and emotional stabilisation with eye movement desensitisation and reprocessing and treatment-as-usual to treatment-as-usual with adults with intellectual disabilities who have post-traumatic stress disorder (the Trauma-AID trial) |
| Countries of Recruitment | England |
| Health Condition(s) or Problem(s) Studied | Post-traumatic Stress Disorder, Intellectual Disabilities |
| Intervention(s) | Active Comparator: Psychological and Emotional Stabilisation and Eye Movement Desensitisation and Reprocessing and Treatment-as-Usual  Comparator: Treatment-as-Usual |
| Key Inclusion and Exclusion Criteria | Participants are eligible to take part in this trial if: (1) they are aged 18 or older, but younger than 66, (2) have a Full Scale IQ <75, (3) meet diagnostic criteria for PTSD, (4) have suffered a major identified trauma at least a year earlier, and (4) are able to communicate using English and have capacity to consent to take part in this clinical trial. Carers are eligible to take part in the trial if: (1) they are aged 18 or older, (2) are a family member or carer of a person with intellectual disability who has consented to take part, and (3) are able to communicate in English.  Those with intellectual disabilities who are: (1) judged to be at high risk or require urgent treatment, (2) currently in therapy and unwilling to intermit, (3) previously completed a course of EMDR, (4) uncontrolled psychosis, (5) unable to complete the outcome assessments, or (6) have a medical condition which, in the opinion of the investigators, could affect the safety of the participant are excluded. |
| Study Type | Interventional phase III; allocation: randomised 1:1 using minimisation; masked assessors; primary purpose: treatment for PTSD |
| Date of first enrolment | 01 Nov 2019 |
| Target Sample Size | 144 adults with intellectual disabilities |
| Recruitment Status | Closed. In follow-up |
| Primary Outcome | Symptoms of post-traumatic stress disorder (Impact of Events Scale – Intellectual Disabilities) |
| Key Secondary Outcomes | Additional mental health problems, participant and carer quality of life, carer burdern. |
