# Supplementary material for: A multicentred two-arm parallel single-blind superiority randomised controlled trial comparing psychological and emotional stabilisation with eye movement desensitisation and reprocessing and treatment-as-usual to treatment-as-usual with adults with intellectual disabilities who have post-traumatic stress disorder (the Trauma-AID trial): protocol
Source: BMJ Open. 2025 Dec 17;15(12):e108818. doi: 10.1136/bmjopen-2025-108818 (PMC12716544; doi:10.1136/bmjopen-2025-108818)
Supplement: Supplementary file 2 [file bmjopen-2025-108818.reviewer_comments.pdf]

PEER REVIEW HISTORY

BMJ Open publishes all reviews undertaken for accepted manuscripts. Reviewers are asked to complete a checklist review form and are provided with free text boxes to elaborate on their assessment. These free text comments are reproduced below.

ARTICLE DETAILS

Title (Provisional)

A multicentred two-arm parallel single-blind superiority randomised controlled trial comparing psychological and emotional stabilisation with eye movement desensitisation and reprocessing and treatment-as-usual to treatment-as-usual with adults with intellectual disabilities who have post-traumatic stress disorder (the Trauma-AID trial): Protocol

Authors

Willner, Paul; Anderson, Pippa; Bisson, Jonathan I; Clifford, Clair; Cooper, Vivien; Farrell, Derek; Harris, Shaun; Hiles, Stephen; Holland, Gail; Hutchings, Hayley Anne; Murphy, Glynis H; Rose, John; Stenfert-Kroese, Biza; Unwin, Gemma L; Watkins, Alan; Willot, Sara; Langdon, Peter

---

VERSION 1 - REVIEW

**Reviewer**	**1**
**Name**	**Cope, Sarah**
**Affiliation**	**South West London and Saint George's Mental Health NHS Trust**
**Date**	**09-Sep-2025**
**COI**	**None**

I was pleased to read this protocol for much-needed research. I have the following minor comments:

- On p. 6, line 36, the authors write "bilateral stimulation is critical to change". I think it would more balanced to write something like "bilateral stimulation is considered critical to change", as research has not definitively shown that bilateral stimulation is the key mechanism of effectiveness.

- On p. 6, when discussing the benefits of a stabilisation phase, I think it would be helpful for the reader to know about competing accounts to this premise, e.g. Ad de Jongh and colleagues work challenging the idea that a stabilisation phase before trauma work is always necessary.

- On p. 17, line 60, there is a typographical error - "analyses" should be "analysed".

VERSION 1 - AUTHOR RESPONSE

Reviewer Comments

1. On p. 6, line 36, the authors write "bilateral stimulation is critical to change". I think it would more balanced to write something like "bilateral stimulation is considered critical to change", as research has not definitively shown that bilateral stimulation is the key mechanism of effectiveness.

We have adopted the suggested text.

2. On p. 6, when discussing the benefits of a stabilisation phase, I think it would be helpful for the reader to know about competing accounts to this premise, e.g. Ad de Jongh and colleagues work challenging the idea that a stabilisation phase before trauma work is always necessary.

We have included a sentence to this effect and cited the suggested author.

3. On p. 17, line 60, there is a typographical error - "analyses" should be "analysed".

This has been fixed.
